# Can Rehabilitation in Nature Improve Self-Perceived Interpersonal Problems? A Matched-Control Study

**DOI:** 10.3390/ijerph19063622

**Published:** 2022-03-18

**Authors:** Martin Mau, Maria L. Vang, Anna Mejldal, Simon Høegmark, Kirsten K. Roessler

**Affiliations:** 1 Department of Psychology, University of Southern Denmark, 5230 Odense, Denmark; mlvang@health.sdu.dk (M.L.V.); shoegmark@health.sdu.dk (S.H.); kroessler@health.sdu.dk (K.K.R.); 2 Health, Social Work and Welfare Research, UCL University College, 5230 Odense, Denmark; 3 Health Sciences Research Centre, UCL University College, 5230 Odense, Denmark; 4 Department of Occupational and Environmental Health, Odense University Hospital, 5000 Odense, Denmark; 5 Open Patient Data Exploratory Network, Odense University Hospital, 5000 Odense, Denmark; amejldal@health.sdu.dk

**Keywords:** nature, rehabilitation, self-perceived interpersonal problems

## Abstract

Self-perceived interpersonal problems are of central concern for researchers and individuals; they are at the basis of psychopathology and cause for subjective distress. In this study, we examine whether a group-based rehabilitation program in nature may reduce self-perceived interpersonal problems in a heterogeneous group of men declining participation in traditional rehabilitation offers. The intervention consisted of weekly meetings in nature, taking place over the course of nine weeks. Through a matched-control study including 114 participants in the intervention group and 39 in a treatment as usual group participating in traditional rehabilitation offers, we found that there was no statistically significant development in self-perceived interpersonal problems in the nature-based rehabilitation offer. Though promising with regards to a number of mental challenges, including relational challenges, nature-based group-rehabilitation may require a more elaborate and thoroughgoing intervention, including e.g., a therapist and more time to be an effective intervention against interpersonal problems. We conclude that perhaps due to the fundamental aspect of self-perceived interpersonal problems, exposure to nature, and being in a group of men in a similar situation for the duration that this intervention lasted, may not be enough to address such underlying perceptions of self.

## 1. Background

Exposure to nature or outdoor environments can have remedying effects on mental health issues and can be an active component in mental health interventions [1]. Ever since the hallmark study by Roger S. Ulrich [2] on the effects of having a hospital window overlooking trees versus having a hospital window with a view to a brick wall among post-operative patients, showing that exposure to nature may reduce for example the use of pain medication, a number of other effects of exposure to nature have been identified. Notably, Kaplan and Kaplan [3] and Ulrich et al. [4] have attempted to explain the beneficial effects of nature on human well-being starting from different perspectives. Kaplan & Kaplan’s [3] theory of Attention Restoration focuses on nature’s influence on cognitive processes, whereas Ulrich et al.’s [4] Stress Reduction Theory focuses on physiological and emotional processes. These basic theories have informed numerous interventions across the past three decades [5], for example focusing on cognitive processes such as working memory [6] and emotional processes, such as self-reported stress and mood [7,8]. Both these theories build in part on a cognitive theoretical background [4,9]. A lesser-studied area of research is self-perceived interpersonal problems, the focus of this study, which is examined using on a measure building on psychodynamic and object relations theory [10].

To harness or capture the benefits of exposure to nature, researchers have examined a number of different, more or less formalized, approaches. “Wilderness therapy” [11] and “nature-assisted therapy” [12] are among the concepts, which have been applied within research. Other studies examine “forest bathing” [13], “nature therapy” [14], or walks taking place in nature [15,16]. Across these types of approaches, nature is used either as the intervention, or as part of an intervention alongside other components such as movement or physical exercise (e.g., walking, running), psychotherapy, or psychotherapeutic exercises (e.g., mindfulness training). Consequently, the beneficial effects of exposure to nature can be studied in isolation, but in practice, exposure to nature is often a part of a larger intervention. One explanation behind this can be that combining exposure to nature with an intervention that promotes engagement with the environment, may enhance the beneficial effects of exposure to nature [17].

### 1.1. A Nature-Based Rehabilitation Program

In this study, we have conducted a group-based rehabilitation program in nature, known as The Wildman Programme [18]. The content and the overall result of this intervention have been described in detail elsewhere [19,20]. Briefly, the program was based on 4 pillars: (1) nature exposure, for example being presented with scenic areas and doing sensory activities, (2) body awareness training, for example, doing breathing exercises, (3) mind relaxation and attention training, for example, meditation, and (4) activities designed to support community spirit, for example, bonfire cooking.

The Wildman Programme was a group-based intervention, designed to accommodate men who did not wish to participate in more traditional rehabilitation programs, and who were challenged by a broad range of mental and somatic problems. This intervention differs from traditional rehabilitation offers by taking place in nature and was designed to appeal to men, who are known to have a high drop-out rate from traditional rehabilitation programs that are conversation-based [21,22,23,24]. Previous research has documented that the program has positive effects on quality of life and stress [19], however, the potential of nature-based interventions to affect interpersonal problems is under-examined, and therefore the focus of the current study.

Being in a natural setting can be socially restoring by providing a different social experience than one would receive in an urban setting [25]. Hypothetically, by changing the setting from participants’ homes or from the municipal institutions where interventions usually take place, people are offered a chance to participate in new social experiences that may change their perception of their social identity. Moreover, this group-based intervention may address negative perceptions of self by helping participants undergo corrective emotional experiences, where new behavior is tested in a more secure environment [26]. Nature may be a suitable setting for conducting rehabilitating interventions more generally [27], and may be used in connection with social activities to enhance and help along with the building of social relationships among at-risk populations [28]. Moreover, one study shows, that exposure to nature may provide a sense of self-transcendence, which may help increase feelings of connectedness to one’s social group [29]. Therefore, it is relevant to examine self-perceived interpersonal problems as part of rehabilitation processes. Rehabilitation in a Danish context is meant to be a cooperative process involving both relatives of the individual and professionals, to help people achieve independence and meaningful life [30,31]. Self-perceived interpersonal problems are associated with independence [10], and social relations in which the person is satisfied with his/her role, are related to meaning in life [32].

Moreover, self-perceived interpersonal problems are, in themselves, unwanted and the cause of subjective distress [33], and can or should be addressed for this reason alone. Moreover, interpersonal problems are a fundamental aspect of psychopathology [34,35]. Interpersonal problems can be both the consequence of, but also the cause of emotional problems [36], for example, depression [37] and anxiety [38]. Self-perceived interpersonal problems may also affect well-being [36]. Believing oneself to be overly cold or intrusive may cause one to devalue oneself. Moreover, it may cause one to change one’s social behavior, perhaps isolating oneself or holding back from sharing thoughts or feelings. This may lead to painful and difficult feelings of loneliness [39], but may also lead to spillover effects on well-being [40]. Self-perceived interpersonal problems have also been used to examine the role of interpersonal problems in a number of other psychological challenges including alcohol abuse [41] and occupational stress [42].

For these reasons, transferring a focus on interpersonal problems into the growing field of interventions in nature is therefore warranted, however, so far this has only happened on rare occasions. Outcomes similar to self-perceived interpersonal problems have been examined before, however. A systematic review of controlled and observational studies examining nature-assisted therapy [12] differentiated between psychological, intellectual, social, and physiological or physical outcomes. Social outcomes included, among others, levels of engagement, behavior, and cooperation. Psychological outcomes included, for example, independency, confidence, and self-efficacy. Regarding both types of outcomes, the review concludes that nature-assisted therapy may lead to beneficial effects, and with regards to the social outcomes specifically, that such interventions may improve, for example, family functioning [12,43]. The here applied measure, focusing on the self-perceived interpersonal problems, lies in the cross-field between social and psychological outcomes. Thus, we do not focus on participants’ behavior, or in any other way how they appear socially. We focus on how they think they behave or appear: how they perceive their own interpersonal problems.

Self-perceived interpersonal problems have previously been modeled based on Sullivan’s interpersonal theory and Leary’s circumplex model and operationalized using the Inventory of Interpersonal Problems (IIP) [34,44]. According to this approach, interpersonal behavior can be approximated using eight categories of self-perceived problematic interpersonal patterns of behavior. These octants are labeled: domineering, intrusive, overly nurturant, exploitable, nonassertive, socially avoidant, cold, and vindictive [45]. These eight specific categories express a combination of two underlying continuums of interpersonal behavior: affiliation, which ranges from hostile to friendly behavior, and power, or dominance, ranging from submissive to dominating behavior [10].

### 1.2. Aim

The aim of this study is to examine whether participation in the Wildman Programme can improve self-perceived interpersonal problems among a heterogeneous group of men suffering from mental health problems or long-term illnesses compared to treatment as usual.

## 2. Materials and Methods

### 2.1. Study Design

The study was a matched-control study [18], in which one group received a rehabilitation intervention taking place in nature (the Wildman Programme), and a control group received treatment as usual: a rehabilitation intervention offered by the municipality, not taking place in nature. The study was designed as a match control study, in which project administrators sought to match the control group with the gender, age, diagnostic presentation and degree of difficulty of the intervention group. The main aim of the intervention was to improve the general self-experienced quality of life of the participants. IIP was measured at baseline (T1), post-intervention (T2), and 6 months post-intervention (T3).

### 2.2. Participants

A total of 114 men participated in the Wildman Programme and 39 men participated in treatment as usual. However, 4 participants in the Wildman Programme did not respond to the IIP and were excluded from the analysis, leaving a total of 110 in the Wildman Programme and 39 in TAU. All of the participants were retired or on long-term sick leave, related to either mental or somatic illness according to the International Classification of Diseases from the American Medical Association (ICD-10-CM) [46]. Participants were either diagnosed with depression (ICD: F32.0) or stress (ICD: F43.8) and/or heart disease (ICD 151.9), cancer or post-cancer (ICD: C80.1 and Z08), type-2 diabetes (ICD: E11), pain (ICD: R52) or chronic obstructive pulmonary disease (ICD: J44.9).

Two project managers, who each had the responsibility of recruiting through one municipality (Svendborg and Faaborg-Midtfyn in Denmark), conducted recruitment to both the intervention and control groups. Contact with potential participants was established through their general practitioner, the job center, or the local healthcare center. Participants in the Wildman Programme had declined to participate in the existing rehabilitation offers.

Before inclusion in the intervention, potential participants were interviewed to determine whether they were motivated and practically able to participate considering e.g., current treatment (see [19]). Participation was voluntary and the Wildman Programme ethically approved the study (ClinicalTrials.gov, NCT-number: 04073524) (accessed 1 December 2021).

### 2.3. The Intervention 

#### The Wildman Programme

The intervention consisted of a nine-week program, where participants met with a health professional and a nature guide with experience with the target group, once a week for three hours. Participants met in groups of 10–15. The intervention took place in nature, and the setting changed over the course of the intervention (e.g., forest, landscape with hills, shore). Participants were also instructed in exercises they could do in between sessions and were encouraged to find a personally supportive natural environment.

The intervention was based on the NBMC method, which consists of four elements: Nature, Body, Mind, and Community. The intervention is described in detail in previous publications on this research project [18,19]. Briefly, the component regarding nature included different experiences with and stories regarding elements of nature including animal and plant life, changing weather conditions, and more broadly, the changing of seasons; the component regarding body included qigong and balance exercises, meant to increase body awareness; the component regarding mind included mindfulness-inspired exercises, meant to give participants a break from ordinary thoughts and worries; lastly, the component regarding community included building a community spirit with a focus on an open, relaxed, supportive and positive atmosphere in the group, for example by conversations around a bonfire.

### 2.4. Treatment as Usual (TAU)

The control group followed the traditional rehabilitation offers, administrated by the municipality. The components of this rehabilitation intervention are physiotherapeutic treatment, mindfulness courses, fitness training, and other traditional rehabilitation initiatives such as psychoeducation, and nutritional counseling. The TAU did not take place in nature.

### 2.5. Outcome: Inventory of Interpersonal Problems (IIP)

The 32-item questionnaire Inventory of Interpersonal Problems (IIP) was used to assess self-perceived interpersonal problems [47]. The IIP consists of eight subscales, each indicating a particular self-perceived interpersonal problem. These subscales are combinations of the IIP’s two main domains, which are labeled dominance and affiliation. The IIP is organized in a circumplex structure [45], where the two main domains are placed on the x and y-axis, thus being orthogonal and often negatively correlated. Consequently, high affiliation will, for example, often be followed by low dominance.

Both a high score and a low score on the two main domains express self-perceived interpersonal problems. At one end, the domain of dominance is characterized by believing oneself to be manipulative and controlling, or by opposite beliefs: to be non-assertive, meaning that it is difficult to make one’s needs known to others. The domain of affiliation is at one end characterized by believing oneself to be overly nurturant, for example, caring too much or trusting too much, or, at its opposite end, believing oneself to be cold, for example being unable to express affection.

Together these two domains comprise the first four subscales (dominance, non-assertive, overly nurturant, and cold). The remaining four subscales are viewed as combinations of these and are labeled intrusive, characterized by being overly self-disclosing and attention-seeking, and having problems being alone, exploitable, characterized by difficulty expressing anger and with being taken advantage of by others, socially avoidant, characterized by having difficulty socializing, feeling anxious and embarrassed in the presence of others, and vindictive, characterized by difficulty trusting others and caring about the needs of others [45]. Each subscale has four items. One item example of the domineering subscale is: “I try to change other people too much”; of the intrusive subscale: “It is hard for me to stay out of other people’s business”; of the overly nurturant subscale: “I put other people’s needs before my own too much”; of the exploitable subscale: “I let other people take advantage of me too much”; of the non-assertive subscale: “It is hard for me to be assertive with another person”; of the socially avoidant subscale: “It is hard for me to socialize with other people”; of the cold subscale: “I keep other people at a distance too much”; and of the vindictive subscale: “I fight with other people too much” [10].

The IIP is scored on a 5-point Likert-like scale ranging from 0 (‘not at all’) to 4 (‘extremely’) [45] where a higher score indicates increasing difficulty regarding the specific interpersonal situation the item relates to. Research supports both the internal consistency, test-retest reliability, and construct validity of the IIP [44,48]. Cronbach’s alpha at baseline for the subscales in the current study ranged from α = 0.49 (Intrusive) to 0.86 (Cold). Please see Table S1 in Supplementary Materials for Cronbach’s alpha for the individual scales. The majority of the scales displayed satisfactory internal consistency (values above 0.7, [49,50]), the subscale for exploitability and intrusiveness displayed suboptimal values. It is possible that the low reliability of the scales may have affected the results.

### 2.6. Statistical Analysis

The range and distribution of socio-demographic variables at baseline across the two study groups were calculated and compared using chi-squared tests for categorical data and t-tests for continuous data. We performed linear mixed model analysis (LMM) for each of the eight IIP subscales and IIP total score to investigate how the scores were influenced by the variables time point (baseline, follow-up at X weeks, and follow-up at Y weeks), group (Wildman Programme or TAU) and the interaction between time point and group. As participants were not randomized into the two groups (Wildman Programme and TAU), differences in change over time from baseline to each follow-up were estimated, compared, and reported.

For each scale, the need for a subject-specific random slope was tested, and 95% CI’s and *p*-values were calculated with a bootstrap process. Assuming the dropout mechanism is missing at random, linear mixed models deal efficiently with missing values due to dropout using the maximum likelihood estimator. All of the statistical significance tests were 2-tailed with α = 0.05. Analyses were conducted using STATA version 16.

## 3. Results

### 3.1. Demographics

At baseline, the percentage of the IIP scales (sub-scale or total score) missing due to nonresponse on single items varied between 0% and 15% for TAU, and 1% and 26% for the Wildman Programme participants. At the first follow-up, the percentage of the IIP scales lost to follow-up or missing due to nonresponse varied between 18% and 31% for TAU, and 26% and 37% for the Wildman Programme participants. At the second follow-up, it varied between 23% and 31% for TAU, and 49% and 58% for the Wildman Programme-participants.

Table 1 displays the characteristics of the sample at baseline. There were no statistically significant differences between the groups on age, educational levels, employment, and cohabitation status, and whether they had children.

Statistically significantly more men in the Wildman Programme group were referred from the job center (n = 38, 35.2%, vs. n = 3, 8.3% in TAU, *p* < 0.001), whereas more men in TAU were referred from their general practitioner (n = 12, 33.3% vs. n = 8, 7.4% in the Wildman Programme-group, *p* < 0.001). However, most of both TAU (58.3%, n = 21) and the the Wildman Programme-group (57.4%, n = 62) were referred from municipal health centers. More men in the Wildman Programme-group reported psychological problems compared to TAU (52.8%, n = 56 vs. 28.9%, n = 11, *p* = 0.044), whereas fewer men reported physical problems in the Wildman Programme-group compared to TAU (57%, n = 61 vs. 75.7%, n = 28, *p* = 0.011). There were no statistically significant differences in whether the men were taking medication (*p* = 0.13), however, statistically, significantly more men in the Wildman Programme-group were in contact with psychiatric services (22%, n = 20 vs. 3%, n = 1, *p* = 0.012), whereas more men in TAU were in treatment through the healthcare system (88.9%, n = 32 vs. 63.1%, n = 65, *p* = 0.004).

### 3.2. Effects of Intervention on Self-Perceived Interpersonal Problems

**Figure 1:**
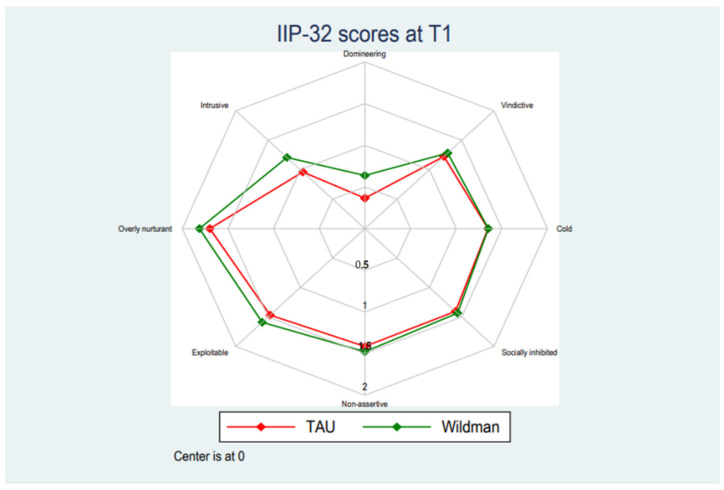
Baseline score pattern for TAU and the Wildman Programme-group.

**Figure 2:**
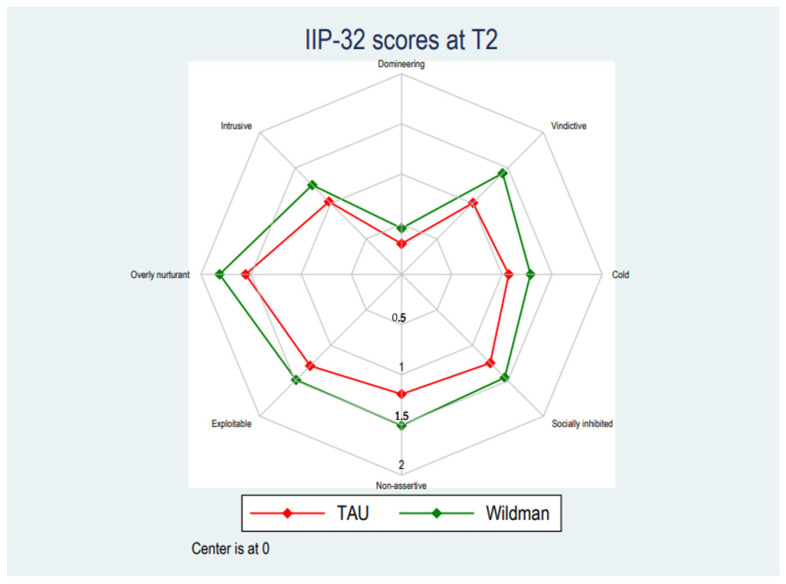
Post-intervention score pattern for TAU and the Wildman Programme-group.

**Figure 3:**
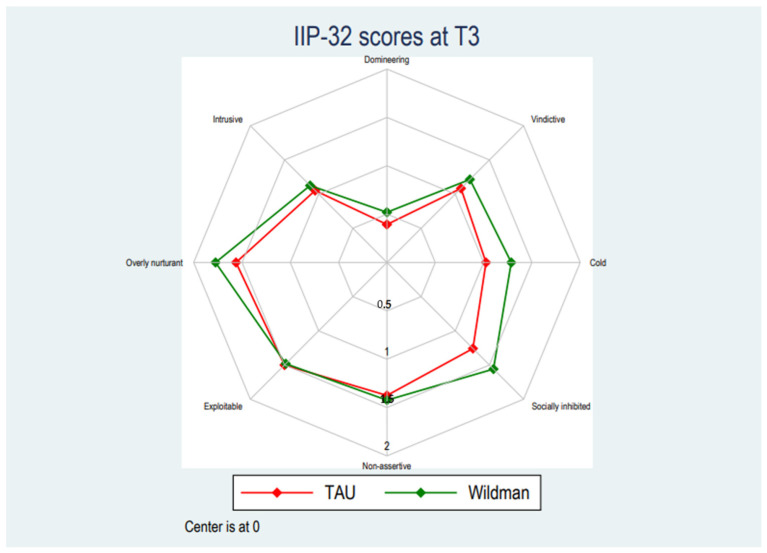
Follow-up score pattern for TAU and the Wildman Programme-group.

Table 2 displays the development in mean scores across baseline (T1), post-treatment (T2), and after 6 months at follow-up (T3). There were no statistically significant differences on neither the total score nor any subscale between pre- and post-treatment compared with the TAU-group. The linear mixed models testing the statistical significance of differences between the groups did not find any statistically significant trends between baseline and post-intervention and baseline and at follow-up (see Figure 1, Figure 2 and Figure 3). The intervention group scored significantly lower on the domineering subscale between baseline and post-intervention (Mean (95% CI): 0.13 [0.01; 0.25], *p* = 0.039), however, statistically significant findings have not been corrected for multiple tests and observing the magnitude of the differences on the domineering subscale, these were small and unlikely to reflect meaningful differences. See Table S2 in Supplementary Materials for full results of the linear mixed model. The number of respondents in the treatment as usual group was between 33–39 at baseline, 27–32 at T2 and 27–30 at T3. The number of respondents in the intervention group was between 81–109 at baseline, 69–81 at T2, and 46–56 at T3. 

## 4. Discussion

The aim of this study was to examine whether participation in the Wildman Programme could improve self-perceived interpersonal problems. The study showed that there were no statistically significant changes during the intervention on either of the IIP subscales nor on the IIP measure as a whole, with one exception being that the Wildman Programme-intervention group significantly improved on the domineering subscale. This effect, however, was not significantly larger when compared to treatment as usual. The overall non-significant findings may be explained by the inabilities of a short-term intervention to facilitate the expected changes. Changing self-perceived interpersonal problems may require a more in-depth and comprehensive intervention ranging over a longer period of time.

Rehabilitation, whether in the traditional or in our different nature-based format, may need more time, a therapist focusing especially on relational aspects present, or otherwise, be improved, to change self-perceived interpersonal problems. Our intervention consisted of meetings in nature, which took place once a week for nine weeks (nine meetings in total). Hypothetically, increasing the frequency of meetings or extending the individual meetings, could have enhanced the effects of the intervention. Had we used a format where participants were continuously in nature for days or weeks, IIP scores could potentially have been influenced. Such longer interventions have been shown to hold great potential with regards to cognitive capacities [51], and may also influence affective states [52].

Moreover, not only the setting but also the activities practiced may have influenced these non-significant findings. The IIP is sensitive to change after psychotherapeutic interventions [34], but as our intervention was not psychotherapeutic as such, we may have been unable to target and change assumptions about oneself in social relations. We hypothesized that participating in a rehabilitation intervention in nature, with other people in similar situations, could provide participants with the opportunity to have different, and positive, social experiences. Although this may still have been the case, we were unable to find an effect of this supposed consequence of the intervention when assessed as changes in the IIP. Non-significant findings are not unique to this study. Other studies trying a apply the IIP to a non-psychotherapeutic intervention have also failed to find the effects of interventions hoped to lead to changes in the IIP [53], indicating that self-perceived interpersonal problems may require an explicit and continuous focus and therapeutic scaffolding to change.

While the Wildman Programme did not prove effective in supporting changes in perceived interpersonal problems, one strength of the program is that it was more appealing to the target group than traditional rehabilitation efforts. This should in itself be taken into account, although neither the intervention nor the TAU led to improvements on our target measure. However, it is also possible that differences between the intervention and control group could have influenced the program’s ability to reduce self-perceived interpersonal problems. Although developments in the levels of self-perceived interpersonal problems were comparable across the intervention and control group, the intervention group more often had psychological challenges. This may have made it more challenging to address the self-perceived interpersonal problems among participants in the intervention group.

## 5. Limitations

When interpreting the results of this study, readers should be attentive to several limitations. There were significant differences between the intervention and control groups, partly reflecting some inadequacies of the matching procedure used in this study. Participants in the intervention group were more often recruited from the job center, more often had psychological challenges, but were significantly less often in treatment (which may also reflect their choice to decline traditional rehabilitation efforts). A randomization procedure would have improved the study, as would larger group sizes. As this study was conducted during the COVID-19 pandemic, it proved difficult to recruit participants. Moreover, our assumption that data were missing at random, may have been optimistic, and even under this assumption, the percentage of missing data was relatively large. This means that the results may have favored treatment as usual, which is the group with the least loss to follow-up, and results may therefore err to the conservative side.

Additionally, nature-based interventions, including this one, are somewhat unclear regarding the effects of different aspects of the intervention. More research is needed to further study the effect and interplay of different aspects of these interventions. The challenges of the current study also reflect an ongoing issue regarding a lack of clarity of the content of interventions in nature entails more generally, perhaps due to the relatively recent discovery of the potentials of exposure to nature for mental health in established health care systems. Differentiating between the effect, or the experiences, associated with, nature and that of, e.g., the therapist, guide, or group has been somewhat overlooked in earlier studies as well as the present.

However, this complexity and the difficulty in differentiating exactly what causes potential effects of nature-based therapy should not discourage us from understanding the potentials of rehabilitation in nature, and this study has provided the field with more indications, that integrating nature in rehabilitation interventions may be used to reach a group, which would otherwise be difficult to include and support through traditional rehabilitation offers.

## 6. Conclusions

The aim of this study was to examine whether a nature-based rehabilitation program could reduce the self-perceived interpersonal problems among a group of men, having declined participation in traditional rehabilitation efforts. Presumably, exposure to nature, and a changing of setting to one, different from the usual municipal rehabilitation setting, could lead to improvements in one’s self-perceived relational difficulties. However, we were unable to detect any meaningful changes in participants’ self-perceived interpersonal problems, with one exception being that the participants in the intervention group significantly improved with regard to feelings of being too domineering. The overall lack of significant findings is valuable knowledge as it illustrates the boundaries of what can be expected of interventions such as this one. A longer intervention, with a more psychotherapeutic content, could potentially have yielded significant effects.

## Figures and Tables

**Table 1:** Baseline demographics and characteristics of the sample.

Characteristic		Treatment as Usual	The Wildman Programme	*p*
Age	Mean (SD)	57.55 (10.70)	54.60 (13.67)	0.23
Education	Lower secondary or less	6 (15.8%)	18 (16.7%)	0.95
	Upper secondary	13 (34.2%)	39 (36.1%)	
	Short cycle tertiary/bachelor	13 (34.2%)	38 (35.2%)	
	Master’s or above	6 (15.8%)	13 (12.0%)	
Employment	Unemployed	3 (7.9%)	20 (18.7%)	0.13
	Employed	13 (34.2%)	21 (19.6%)	
	Flex/ressource	1 (2.6%)	12 (11.2%)	
	Retired	12 (31.6%)	28 (26.2%)	
	Other	9 (23.7%)	26 (24.3%)	
Marital status	Alone	8 (21.1%)	28 (25.7%)	0.57
	Cohabiting	30 (78.9%)	81 (74.3%)	
Parental status	Yes	32 (84.2%)	91 (83.5%)	0.92
	No	6 (15.8%)	18 (16.5%)	

**Table 2:** Differences between mean-scores at baseline, post-treatment, and follow-up.

	Baseline (T1)			Post-Treatment (T2)			Follow-Up (T3)		
IIP Subscale	TAU	The Wildman Programme	*p*	TAU	The Wildman Programme	*p*	TAU	The Wildman Programme	*p*
Domineering	**0.37 (0.55)**	**0.64 (0.64)**	**0.02**	0.30 (0.46)	0.46 (0.57)	0.17	0.39 (0.34)	0.52 (0.57)	0.29
Vindictive	1.22 (1.09)	1.28 (1.00)	0.76	1.01 (1.07)	1.42 (1.11)	0.072	1.08 (0.97)	1.21 (1.10)	0.59
Cold	1.35 (1.15)	1.35 (0.93)	0.99	1.07 (0.84)	1.29 (0.99)	0.30	1.02 (0.92)	1.29 (1.14)	0.28
Socially inhibited	1.40 (1.02)	1.44 (0.97)	0.87	1.25 (0.94)	1.45 (0.98)	0.34	1.26 (1.04)	1.56 (1.24)	0.28
Non-assertive	1.41 (0.95)	1.48 (0.96)	0.73	1.19 (1.00)	1.51 (0.91)	0.12	1.38 (1.05)	1.42 (0.93)	0.82
Exploitable	1.47 (0.73)	1.59 (0.80)	0.40	1.29 (0.78)	1.49 (0.70)	0.19	1.50 (0.80)	1.48 (0.68)	0.91
Overly nurturant	1.70 (1.00)	1.81 (0.86)	0.50	1.55 (0.79)	1.81 (0.79)	0.12	1.56 (0.84)	1.77 (0.86)	0.29
Intrusive	0.96 (0.65)	1.21 (0.73)	0.07	1.02 (0.83)	1.26 (0.74)	0.15	1.05 (0.67)	1.13 (0.70)	0.65
Total	1.25 (0.70)	1.37 (0.55)	0.34	1.11 (0.64)	1.30 (0.57)	0.17	1.14 (0.54)	1.26 (0.63)	0.39

Note: Values are M (SD). TAU n = 39, Wildman Programme n = 110. Statistically significant differences are highlighted in bold.

## Data Availability

The data presented in this study are according to Danish law not available for sharing.

## Supplementary Materials

The following supporting information can be downloaded at: https://www.mdpi.com/article/10.3390/ijerph19063622/s1, Table S1: Cronbach’s alpha values for the subscales of IIP, Table S2. Difference scores between TAU and the Wildman Programme at baseline, post-treatment and follow-up.
