# Supplementary material for: Can Rehabilitation in Nature Improve Self-Perceived Interpersonal Problems? A Matched-Control Study
Source: Int J Environ Res Public Health. 2022 Mar 18;19(6):3622. doi: 10.3390/ijerph19063622 (PMC8949583; doi:10.3390/ijerph19063622)
Supplement: Supplementary file 1 [file ijerph-19-03622-s001.zip › ijerph-1589585-supplementary.pdf]

Supplementary Table S1: Cronbach's alpha values for the subscales of IIP.

Scale	Alpha
Domineering	0.76
Vindictive	0.85
Cold	0.86
Socially inhibited	0.81
Non-assertive	0.83
Exploitable	0.58
Overly nurturant	0.79
Intrusive	0.49

Supplementary Table S2. Difference scores between TAU and the Wildman Programme at baseline, post-treatment and follow-up.

Outcome	Change	TAU		The Wildman Programme		Difference between TAU and the Wildman Programme	
		Mean (95 % CI)	p	Mean (95 % CI)	p	beta (95 % CI)	p
Domineering	T1 to T2	0.01 (-0.1; 0.13)	0.818	**0.13 (0.01;0.25)**	**0.039**	0.11 (-0.06;0.29)	0.202
	T1 to T3	-0.11 (-0.24; 0.03)	0.112	0.10 (-0.04; 0.25)	0.163	**0.21 (0.02;0.41)**	**0.035**
Vindictive	T1 to T2	0.15 (-0.12; 0.43)	0.279	-0.16 (-0.42; 0.09)	0.210	-0.32 (-0.7;0.07)	0.104
	T1 to T3	0.07 (-0.24; 0.38)	0.656	0.11 (-0.20; 0.43)	0.482	0.04 (-0.44;0.18)	0.865
Cold	T1 to T2	0.20 (-0.04; 0.45)	0.100	0.08 (-0.09; 0.25)	0.367	-0.13 (-0.44;0.18)	0.426
	T1 to T3	0.28 (-0.08; 0,63)	0.126	0.10 (-0.19; 0.39)	0.488	-0.18 (-0.60;0.24)	0.412
Socially avoidant	T1 to T2	0.18 (-0.18; 0.54)	0.332	0.03 (-0.15; 0.20)	0.767	-0.15 (-0.55;0.24)	0.453
	T1 to T3	0.18 (-0.18; 0.54)	0.426	-0.08 (-0.40;0.24)	0.632	-0.20 (-0.61;0.20)	0.331
Nonassertive	T1 to T2	0.16 (-0.17; 0.50)	0.330	-0.01 (-0.20;0.18)	0.941	-0.17 (-0.59;0.24)	0.416
	T1 to T3	-0.01 (-0.39; 0.37)	0.976	0.11 (-0.18;0.40)	0.461	0.12 (-0.33;0.56)	0.607
Exploitable	T1 to T2	0.20 (-0.08; 0.48)	0.163	0.10 (-0.04; 0.24)	0.167	-0.10 (-0.43;0.23)	0.552
	T1 to T3	-0.09 (-0.39; 0.37)	0.568	0.15 (-0.05; 0.35)	0.150	0.24 (-0.13;0.60)	0.205
Overly nurturant	T1 to T2	0.17 (-0.08; 0.43)	0.178	0.03 (-0.11; 0.17)	0.674	-0.14 (-0.45;0.16)	0.354
	T1 to T3	0.11 (-0.11; 0.33)	0.313	0.07 (-0.13;0.26)	0.511	-0.05 (-0.34;0.25)	0.754
Intrusive	T1 to T2	-0.11 (-0.32; 0.09)	0.290	-0.09 (-0.14;0.18)	0.210	0.02 (-0.23;0.28)	0.870
	T1 to T3	-0.12 (-0.31; 0.08)	0.234	0.02 (-0.14;0.18)	0.794	0.14 (-0.10;0.38)	0.250
Total score	T1 to T2	0.09 (-0.06; 0.25)	0.244	0.07 (-0.06; 0.20)	0.298	-0.02 (-0.24;0.19)	0.833
	T1 to T3	0.07 (-0.14; 0.27)	0.536	0.13 (-0.05;0.30)	0.163	0.06 (-0.23;0.35)	0.676

Note: T1: Baseline. T2: Post-intervention. T3: Follow-up. TAU: Treatment as Usual. beta values are unstandardized. Statistically significant differences are highlighted in bold, however, these have not been corrected for multiple tests.
